# Task-Specific Adaptive Differential Privacy Method for Structured Data

**DOI:** 10.3390/s23041980

**Published:** 2023-02-10

**Authors:** Assem Utaliyeva, Jinmyeong Shin, Yoon-Ho Choi

**Affiliations:** School of Computer Science and Engineering, Pusan National University, Busan 609-735, Republic of Korea

**Keywords:** differential privacy, machine learning, privacy-preserving

## Abstract

Data are needed to train machine learning (ML) algorithms, and in many cases often include private datasets that contain sensitive information. To preserve the privacy of data used while training ML algorithms, computer scientists have widely deployed anonymization techniques. These anonymization techniques have been widely used but are not foolproof. Many studies showed that ML models using anonymization techniques are vulnerable to various privacy attacks willing to expose sensitive information. As a privacy-preserving machine learning (PPML) technique that protects private data with sensitive information in ML, we propose a new task-specific adaptive differential privacy (DP) technique for structured data. The main idea of the proposed DP method is to adaptively calibrate the amount and distribution of random noise applied to each attribute according to the feature importance for the specific tasks of ML models and different types of data. From experimental results under various datasets, tasks of ML models, different DP mechanisms, and so on, we evaluate the effectiveness of the proposed task-specific adaptive DP method. Thus, we show that the proposed task-specific adaptive DP technique satisfies the model-agnostic property to be applied to a wide range of ML tasks and various types of data while resolving the privacy–utility trade-off problem.

## 1. Introduction

Data are needed to train machine learning (ML) algorithms, and in many cases often include private datasets that contain sensitive information. Sensitive information is a subset of personal information subject to a higher level of privacy protection than other personal information. As Federal Trade Commission (FTC) imposed USD 5 billion fine on Facebook in 2019 [1], the penalties for non-compliance with privacy laws while handling of personal data can be severe due to the increasing array of regulations. To comply with privacy laws regulating personally identifiable information and preserve the privacy of data used while training ML algorithms, computer scientists have widely deployed anonymization techniques such as *k*-anonymity [2,3], *l*-diversity [4], *t*-closeness [5] and so on.

These anonymization techniques have been widely used but are not foolproof. One factor of concern is that data scientists want a dataset that is as big as possible in ML. However, the bigger the size of the dataset is, the more likely sensitive information can be identified from it. Furthermore, another factor of concern is that these anonymization techniques should not only protect data where sensitive information is identified from the dataset, but also data where sensitive information is identifiablefrom the correlation of multiple datasets. However, many studies showed that ML models are vulnerable to an increasing array of various privacy attacks willing to expose an individual [6,7].

To overcome such concerns for various anonymization techniques and protect private data with sensitive information in ML, privacy-preserving machine learning (PPML) techniques have been recently developed using cryptography and statistics. One promising approach using cryptography is the homomorphic encryption technique, which allows ML models computed on ciphertexts to generate the same results as that ML model computed on plaintexts. Another approach using statistics is differential privacy (DP). A PPML algorithm is said to be *differentially private* in the computation, where the presence or absence of a specific individual’s information in the dataset cannot be identified from the output using the dataset with random noise generated according to a carefully chosen distribution.

Current research works on DP techniques in ML are mainly categorized into differentially private model modification techniques; and differentially private data publishing techniques. Differentially private model modification techniques consider how to safely share the ML model among separate entities against the leakage of hyperparameters to attackers. That is, differentially private model modification techniques focus on protecting not the data itself but hyperparameters of the ML model against various privacy attacks such as model inversion [8] and model stealing attacks [9]. On the other hand, differentially private data publishing techniques consider how to generate the perturbed data itself in order to share the private data among separate entities for ML. Since we are interested in protecting the individual information in the data for ML, we focus on differentially private data publishing techniques.

Differentially private data publishing techniques have been commonly designed using marginal distributions (MD) [10,11,12] and generative adversarial networks (GAN) [13,14]. Even though such techniques successfully generate private data whose distribution is similar to the original data distribution, their usage is constrained due to the following issues:

- No adaptive noise generation for the structured data: When adding the singular distribution of noise into a data record, the utility of data may increase or decrease according to the types of data. This is because continuous numerical values with the additive noise mimic the same distribution of the original data, while discrete and ordinal values with the additive noise show a significantly different distribution from the original data. Thus, we need adaptive noise generation techniques for structured data while keeping the same distribution as the original data distribution.
- No attribute-wise adaptive noise generation for given tasks of ML models: Let us consider that we add the same amount of noise into attributes with high or low relevance to ML tasks. Since attributes with higher relevance to ML tasks include the same amount of random perturbation as the attributes with lower relevance, the utility of data decreases. That is, when the same amount of noise for each attribute is given, the relevance of each attribute to given tasks of ML models as well as the utility of data can change. To preserve the higher utility of data with random perturbation for given tasks of ML models, we need to add different amounts of noise into data attributes according to feature importance.
- Privacy and utility trade-off: In the context of DP, the privacy and utility trade-off problem is represented as the trade-off issue between privacy parameter  ϵ and the strength of privacy. Here, privacy parameter ϵ is a metric of privacy loss at a differential change in data, i.e., adding or removing 1 entry. The smaller ϵ is, the stronger the privacy strength is and the lower the data utility is. On the other hand, the larger the ϵ value is, the weaker the privacy strength is and the higher the data utility is. This trade-off issue indicates the need of designing effective differentially private data publishing techniques which guarantee lower privacy parameter ϵ while keeping higher utility of data.

To resolve the issues described above, we propose a new task-specific adaptive DP technique for structured data. To resolve the first issue, we generate noise using differentially private mechanisms, each of which fits different types of data. To resolve the second issue, we adaptively calibrate the amount of random noise following the feature importance for the given tasks of ML models. Finally, by combining the above adaptive DP techniques, we resolve the third issue, i.e., the privacy–utility trade-off problem.

The main idea of the proposed DP method is to adaptively calibrate the amount and distribution of random noise applied to each attribute according to the feature importance for the specific tasks of ML models and different types of data. Thus, the proposed task-specific adaptive DP technique satisfies the model-agnostic property to be applied to a wide range of ML tasks and various types of data. Furthermore, we note that while preserving higher utility and privacy guarantee under a practical privacy attack, adaptive addition of random noise to each attribute using the proposed technique provides robustness against Attribute Inference Attack (AIA) [6]. In AIA, the adversary with partial records of data and access to the model infers sensitive information in data.

The main contributions can be summarized as follows. First, we introduce a new task-specific adaptive DP method for structured data. The proposed DP method resolves the privacy–utility trade-off problem while keeping the same distribution as the original data distribution and preserving the higher utility of data with random perturbation for different tasks of ML models. Second, to effectively resolve the attribute-wise adaptive noise generation problem, we introduce a new sensitivity measure, called *adaptive sensitivity*, while the traditional sensitivity measure calibrates random noise only in the context of the presence or absence of a record, *adaptive sensitivity* calibrates random noise in the context of the feature importance as well as the presence or absence of a record. Thus, the attributes with higher relevance to ML tasks include fewer random perturbations to preserve the higher utility of the data. Third, we experimentally show that the proposed task-specific adaptive DP method is applicable to various datasets, whose record includes different types of data.

The rest of the paper is organized as follows. After describing the background information to understand the proposed task-specific adaptive DP method in Section 2, we overview the related works in Section 3. We describe the operational details of the proposed DP method in Section 4. In Section 5, we show the experimental results with various datasets for different ML models. Finally, we summarize the paper in Section 6.

## 2. Preliminaries

In this section, we introduce the definition of the sensitivity, differentially private mechanisms, and properties of DP to understand the proposed DP method.

### 2.1. Record Sensitivity

We determine that a deterministic function or mechanism *f* (e.g., the database query) satisfies DP if for all neighboring datasets $D_{1}$ and $D_{2}$, which differ in the data of a single individual, and all possible outputs *S*,

$$\frac{Pr[f\left(D_{1}\right)=S]}{Pr[f\left(D_{2}\right)=S]}\leq e^{\epsilon },$$

where privacy parameter ϵ tunes the amount of privacy guarantee.

To hide a single data record for a deterministic function *f*, DP adds different distributions of random noise or different amounts of random noise into the input of a function *f*. In general, the sensitivity of a function, called global sensitivity, reflects that the amount of the function’s output will change when its input changes.

Since the amount of random noise is calibrated by tuning the value of privacy parameter ϵ, sensitivity is defined as follows. That is, for a function f:D→R, which maps dataset (*D*) to real number (*R*), the sensitivity of a function *f* is defined into the maximum difference between $f(D_{1})$ and $f(D_{2})$ as follows:

$$\Delta f=\max_{D_{1},D_{2}:d\left(D_{1},D_{2}\right)\leq 1}∥f\left(D_{1}\right)-f\left(D_{2}\right)∥,$$

where ∥·∥ refers to the norm, i.e., either $l_{1}$ or $l_{2}$ norm, and the distance $d(D_{1},D_{2})$ between any two datasets $D_{1}$ and $D_{2}$ differs at most in the data of a single individual.

Note that since the data of a single individual is the data of a single record in the structured dataset, sensitivity denotes *record sensitivity*. However, to resolve the attribute-wise adaptive noise generation problem, the sensitivity of the proposed task-specific adaptive DP method needs to be computed by considering the feature importance of each attribute as well.

### 2.2. Differentially Private Mechanisms

To satisfy DP, different noise addition techniques, called *differentially private mechanisms*, generates various random noise distributions and different amount of random noise. *Differentially private mechanisms* are classified into two categories: (1) output perturbation mechanisms; and (2) sampling from distribution mechanisms.

- *Output perturbation mechanisms* guarantees DP by adding noise with random distributions directly to *continuous* data.The Laplace mechanism [15] is a representative output perturbation mechanism, where random noise drawn from Laplace random distribution is added to the numerical value as follows:
  $$M_{L}\left(D,f\left(\cdot \right),\epsilon \right)=f\left(D\right)+Lap(\frac{\Delta f}{\epsilon }),$$
  where f(D) is the original numerical value, Lap is the probability density function of Laplace random distribution, ϵ is the privacy parameter, and Δf is the sensitivity.
- *Sampling from distribution mechanism* guarantees DP by sampling from a problem-dependent family of elements with random exponential probability scaled by privacy parameter ϵ and sensitivity. Different from output perturbation mechanisms, the randomized output is always a member of a problem-dependent family. As a representative mechanism, the Exponential mechanism guarantees DP by outputting r∈R with the following probability:
  $$\propto exp(\frac{\epsilon f(D,r)}{2\Delta f}),$$
  where *r* is the member of the set *R* that is a set of possible values. Thus, such a mechanism guarantees DP for non-continuous values.

Since real-life datasets commonly contain a variety of attribute types, random noise generated from only a singular mechanism leads to low-quality data generation. To consider various types of attributes and their feature importance, the proposed task-specific adaptive DP method adopts adaptive differentially private mechanisms.

### 2.3. DP Properties

Due to the following properties of DP, differentially private mechanisms that satisfy DP offer a strong privacy guarantee.

- *Post-processing* [16] property states that if F(x) satisfies ϵ-DP, then for any deterministic or randomized operation *g* on F(x), g(F(X)) satisfies ϵ-DP.
- *Composition* [16] property states a combination of differentially private outputs, whose types are sequential composition and *parallel composition*.
  - *Parallel composition* bounds the total privacy cost of multiple data releases by splitting the dataset into disjoint chunks and running a differentially private mechanism on each chunk separately. That is, *parallel composition* implies that when applying $\epsilon_{i}$-DP to disjoint chunks of data, the combination of all of the results satisfies $\epsilon =max(\epsilon_{i})$-DP.

Based on *post-processing* property and *parallel composition*, data generated using the proposed task-specific adaptive DP method satisfies DP.

## 3. Related Work

Differentially private data publishing techniques add random perturbation into data to guarantee DP. Since the proposed task-specific adaptive DP method belongs to differentially private data publishing techniques, we overview well-known differentially private data publishing methods. Differentially private data publishing methods are mainly categorized into MD-based methods and GAN-based methods.

As a representative MD-based method, N. Mohammed et al. proposed DiffGen [17], which added random noise to one-way marginals. However, DiffGen did not capture an important correlation between attributes. Thus, DiffGen generated lower utility data. To overcome the limitation of DiffGen, Z.Zhang et al. proposed PrivSyn [11], which captured the correlation between attributes by considering three-way marginals. Furthermore, K.Cai et al. proposed PrivMRF [18] that used a set of low-level marginals to construct a Markov Random Field to model the internal correlation in the dataset. Another method to generate differentially private data was introduced by R.McKenna et al., a PrivPGM [19] that measured low-level marginals with a noise mechanism. Different from the other methods, J. Zhang et al. proposed PrivBayes [10], which used Bayesian networks while generating differentially private data. Furthermore, since MD-based methods require the computation of marginals and can require the construction of graphical models, these methods cause high computational complexity.

As a representative GAN-based method, L.Xie et al. proposed DPGAN [14], which added noise to the gradients of the generator by clipping weights. On the other hand, X.Zhang et al. proposed dp-gan [20], and Y.Liu et al. proposed PPGAN [21] which added noise to the discriminator using differentially private stochastic gradient descent (DPSGD) optimizer. Furthermore, J.Jordon et al. proposed PATE-GAN [13], which integrates a DP method, called PATE [22], into a discriminator. As a variant of GAN-based methods, DPCGAN [23] considering conditional GAN model also was proposed. Since GAN-based methods attempt to generate synthetic data that is similar to the original data distribution, differentially private components of the GAN-based model can have larger values of privacy parameter ϵ, thus, providing a lower level of privacy guarantee.

Privacy–utility trade-off is a common problem of both MD-based methods and GAN-based methods. To generate high utility data whose distribution is similar to the original data distribution, these DP methods guarantee DP with large values of ϵ. As described in [7], large values of ϵ cause to expose sensitive information under privacy attacks targeting data. On the other hand, while generating data with high privacy guarantees, these DP methods decrease the utility of data. For example, noise added to the data for preventing privacy attacks leads to incorrect predictions [6]. Furthermore, we note that none of the MD-based and GAN-based methods considers the task of ML models that will use these differentially private data, as well as different types of data contained in the dataset. Thus, these DP methods cause low utility of data. As a new differentially private data publishing method that covers the problems mentioned above, we propose a new task-specific adaptive DP method for structured data.

## 4. Task-Specific Adaptive DP

After introducing the targeted attribute inference attack, we describe the detailed operation of the proposed task-specific adaptive DP method for structured data and provide an operational example. In Table 1, we summarize symbolic notations to understand the operation of the proposed DP method.

### 4.1. Attribute Inference Attack

Since data used in various tasks of ML models are vulnerable to privacy attacks, sensitive information is exposed to various privacy attacks. Let us overview the operation of the traditional AIA [6], while the fitted ML model is shared with an untrusted third party without disclosure of train data, the adversary gains oracle access to the ML model. Furthermore, when an adversary has a partial target record of the victim, i.e., sensitive data or attributes, An adversary runs AIA by continuously querying the ML model with a large set of sibling records. Sibling records are created by fulfilling partial target records with a set of possible values for unknown attributes. As a result of AIA, an adversary estimates unknown sensitive attributes.

**Figure 1:**
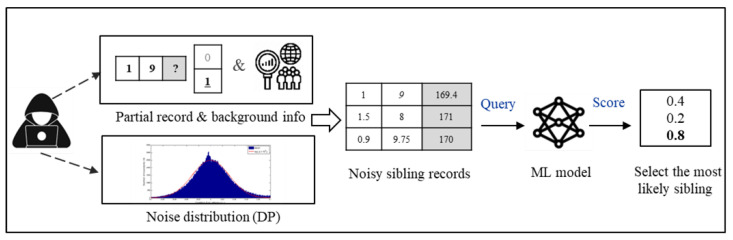
Modified AIA.

In this paper, we consider a modified AIA as shown in Figure 1, where the adversary is aware of the exact distribution of random perturbations applied to data as well. Compared to traditional AIA, modified AIA causes less effectiveness of existing DP methods applied to data.

### 4.2. Overall Workflow

**Figure 2:**
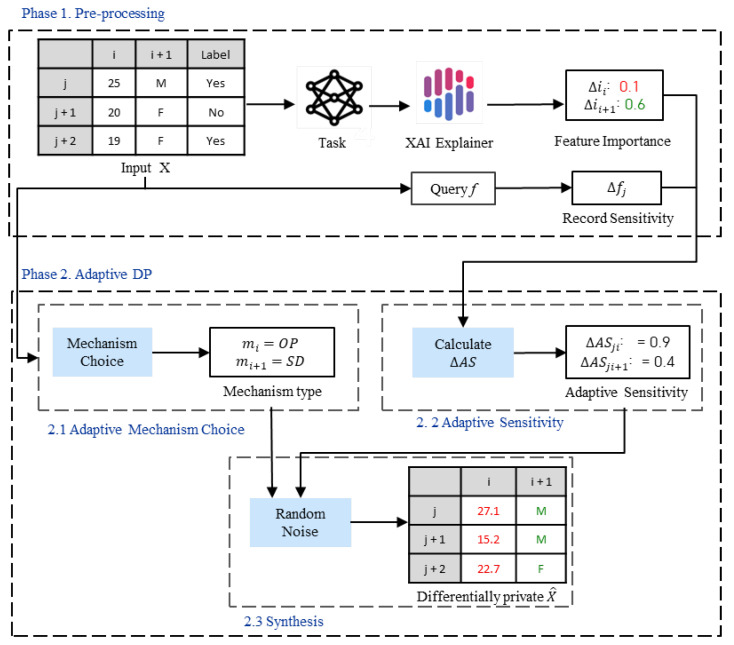
Overall operation of the proposed DP method.

Let us overview the overall workflow of the proposed DP method. As shown in Figure 2 the proposed DP method consists of two phases: pre-processing; and adaptive DP.

- **Phase 1. Pre-processing** is responsible for task initialization. The task of the ML model is manually defined, trained, and fitted according to the preferences or any other nuances of how the data will be used. Next, we estimate the feature importance of each attribute Δi with respect to the task of the ML model using the model interpretation technique. Thus, we are able to capture the subtle impact of a specific model on the data interpretation. Last, but not least, we calculate the record sensitivity Δf according to the task of the ML model.
- **Phase 2. Adaptive DP** is responsible for the generation and addition of adaptive noise to data. Here, adaptive noise generation aims to provide tailored random noise generation according to the specific task of the ML model and data types. In other words, the higher the feature importance, the less noise is added to the feature. The lower the feature importance, the more noise is added to the feature. Furthermore, by adaptively choosing the differentially private mechanism according to data type, the proposed method aims to preserve the distribution of noisy data close to the distribution of original data. We implement adaptive DP techniques and add generated noise to data. Phase 2 consists of the following steps:
  - *Step 2.1 Adaptive mechanism choice* chooses the appropriate noise distribution for each data type.
  - *Step 2.2 Adaptive sensitivity* calibrates noise according to the feature importance of each attribute.
  - *Step 2.3 Synthesis* adds generated random noise to data.

In Figure 2, we show an example that describes the overall operation of the proposed task-specific adaptive DP method for structured data. In phase 1, for the given input data *X*, feature importance Δi for each attribute *i* and record sensitivity $\Delta f_{j}$ for the *j*th record are computed. In phase 2, after we choose the differential private mechanism and calculate adaptive sensitivity using the given Δi and $\Delta f_{j}$, we generate the noisy data $\bar{X}$.

### 4.3. Detailed Operation

Now, let us overview the detailed operation of each phase of the proposed task-specific adaptive DP method.

#### 4.3.1. Phase 1. Pre-Processing

In Algorithm 1, we show the operational steps of pre-processing phase. First, we train a certain ML model *M*, which specifies the task of the ML model, with original data. Second, to estimate the importance of each feature according to the given task *M*, we use the eXplainable Artificial Intelligence (XAI) technique that interprets the output of any ML model. Namely, we use SHapley Additive exPlanations (SHAP), a representative XAI technique, which is a game theoretic approach that uses Shapley values to explain the model decisions. As a result of SHAP on the model *M*, we estimate the feature importance Δi of an attribute *i*. The more relevant the feature is, the higher Δi is. Since Δi of an attribute, *i* is measured relative, the total sum of all Δis is equal to 1. Next, we calculate the record sensitivity Δf of a function *f* according to the concept of DP. Since the time complexity of the Algorithm 1 is linear O(*n*), the execution time increases as the input size *n* increases.   
**Algorithm 1:** Pre-processing.
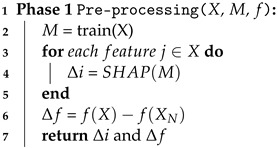


#### 4.3.2. Phase 2. Adaptive DP

Let us describe the operational details of the adaptive DP phase based on the following three steps: *step 2.1 Adaptive mechanism choice*; *step 2.2 Adaptive sensitivity*; and *step 2.3 Synthesis*. Let us overview details of each step in the following subsections.

#### 4.3.3. Step 2.1 Adaptive Mechanism Choice

In this step, we choose appropriate random noise distributions according to various types of data. That is, to add different noise distributions to data, we generate noisy data, which is generated using different differentially private mechanisms for each attribute according to its data type.

As shown in Algorithm 2, we choose differentially private mechanism type *m*, which generates appropriate noise distribution according to the type of data. That is, as mentioned in Section 2, for continuous attributes, output perturbation mechanisms add noise drawn from Laplace or Gaussian distributions. For non-continuous attributes, sampling from distribution mechanisms adds noise drawn from Exponential distribution. Since the efficiency of the Algorithm 2 depends on the size of the dataset, the time complexity is O(*n*).
**Algorithm 2:** Adaptive Mechanism Choice.
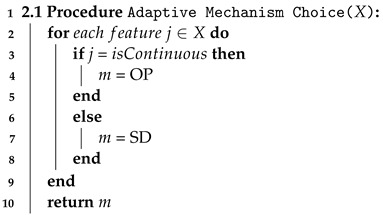


From the perspective of data utility, the adaptive addition of noise for structured data increases data utility by preserving the original distribution for each type of data. On the other hand, from the perspective of data privacy, the adaptive addition of noise for structured data decreases the chances to infer sensitive information under the modified AIA scenario. This is because after random noise generated from different random distributions is added to data, the adversary cannot mimic the random noise distribution added to data due to the properties of DP.

#### 4.3.4. Step 2.2 Adaptive Sensitivity

In the adaptive sensitivity step, we calibrate the amount of random noise according to the feature importance of each attribute Δi. In Algorithm 3, we show the operational steps to calculate *Adaptive sensitivity* for each data element. Therefore, the time complexity of Algorithm 3 is also linear O(*n*), which depends on the input size *n*.   
**Algorithm 3:** Adaptive Sensitivity.
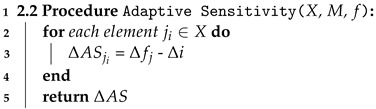


Let us remind that in DP, noise is calibrated according to privacy parameter ϵ and record sensitivity Δf in general. To generate noise while considering the feature importance of each attribute as well, we need a new metric, called *adaptive sensitivity* (*AS*), which combines both record sensitivity Δf and feature importance Δi. The adaptive sensitivity of an element $j_{i}$ in data are calculated in conjunction with the feature importance of an attribute *i* and regular sensitivity of record *j* as follows:

$$\Delta AS_{j_{i}}=\Delta f_{j}-\Delta i.$$.

By adding less noise for relevant attributes with high Δi, $\Delta AS_{j_{i}}$ becomes smaller than $\Delta f_{j}$. Furthermore, by adding relatively large noise for irrelevant attributes with low Δi, $\Delta AS_{j_{i}}$ becomes close to $\Delta f_{j}$. Note that $\Delta AS_{j_{i}}$ cannot be a negative value and the sum of all Δis is equal to 1. Thus, the minimum value of $\Delta f_{j}$ also equals 1.

From the perspective of data utility, attribute-wise adaptive addition of random noise preserves data utility in the context of ML task *M*. From the perspective of data privacy, attribute-wise addition of random noise decreases the chances to infer sensitive information in modified AIA scenarios. This is because different amounts of noise are added to attributes and thus, an adversary cannot mimic the random noise distribution added to data due to the properties of DP.

#### 4.3.5. Step 2.3 Synthesis

In the synthesis step, we generate differentially private random noise with privacy parameter ϵ while varying mechanism type *m*, and adaptive sensitivity $\Delta AS_{j_{i}}$ of an element $j_{i}$. After adding noise to each data element $i_{j}$, we generate differentially private data denoted by $\hat{X}$.

Algorithm 4 shows how to generate differentially private data $\hat{X}$ according to values of ϵ, *m* and $\Delta AS_{j_{i}}$. When mechanism type *m* is output perturbation (OP), such as Gaussian and Laplace differentially private mechanism, we add random noise to an element $j_{i}$ depending on values of $\Delta AS_{j_{i}}$ and ϵ. Specifically, in the Algorithm 4, we assume that OP is the Laplace mechanism. Thus, we add random noise drawn from the Laplace distribution calibrated as $Lap(\frac{\Delta AS_{j_{i}}}{\epsilon })$. When mechanism type *m* is sampling from distribution (SD), we generate differentially private data using sampling from distribution mechanisms that are carefully designed scoring functions of Exponential mechanism depending on values of $\Delta AS_{j_{i}}$ and ϵ. In line 9, we sample the element with exponential probability calibrated using the privacy parameter ϵ, the utility function that is query *f*, and novel adaptive sensitivity $\Delta AS_{j_{i}}$. Therefore, the time complexity of the Algorithm 4 is linear O(*n*), which depends on the input size *n*. As a result, we generate differentially private data $\hat{X}$ that guarantees both high data utility and data privacy.
**Algorithm 4:** Synthesis.
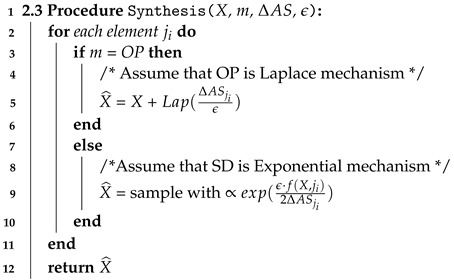


### 4.4. Example

**Figure 3:**
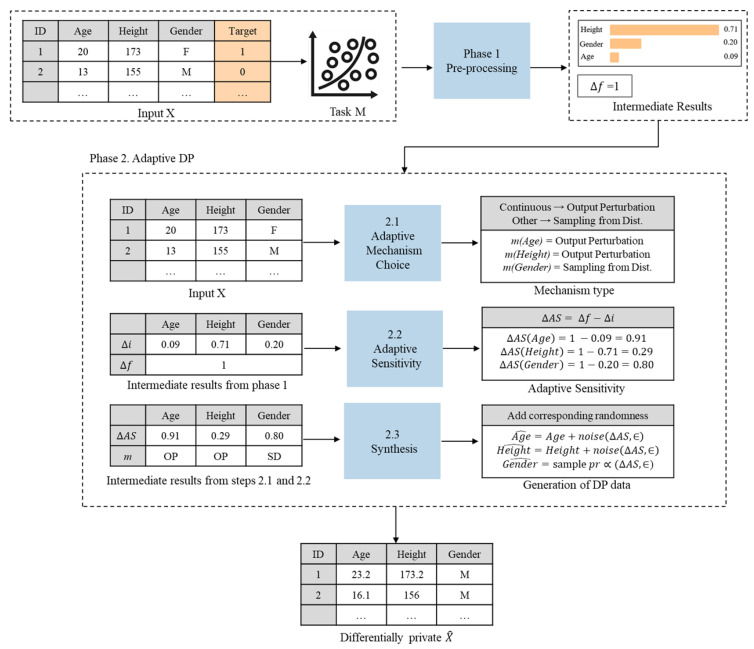
Operational example of the proposed task-specific adaptive DP method.

Let us consider the operational example of the proposed task-specific adaptive DP method. As shown in Figure 3, we assume that structured data *X* consists of *N* instances with 3 attributes whose types of data are different. That is, *X* includes two continuous attributes *Age* and *Height* as well as a categorical attribute *Gender*.

In phase 1. pre-processing, we specify the task of the ML model. That is, we train and fit a certain ML model *M* with original data *X*. The accuracy of ML model *M* on previously unseen original test data are referred to as baseline accuracy, which is used for evaluation purposes. Here, let us assume that baseline accuracy was 97.0%. To calculate record sensitivity $\Delta f_{j}$, we assume that the query $f_{j}$ returns each element in the dataset, and thus, the absence or presence of a single data can result in $\Delta f_{j}$ = 1.

To calculate feature importance Δi, we run an XAI explainer, called SHAP, on model *M* and test input. SHAP ranks input features according to their relative importance scores in descending order, and after normalization total sum of all scores equals 1.

In this example, attribute *Height* has the highest feature importance score Δi equal to 0.71, attributes *Gender* and *Age* have less impact because values of Δi equals to 0.20 and 0.09, respectively.

Next, let us overview the operation of phase 2. adaptive DP. Let us remind that in step 2.1 Adaptive mechanism choice, we choose appropriate differentially private mechanisms to add different noise distributions according to the type of data. That is, output perturbation mechanisms are suitable for continuous attributes *Height* and *Age* while sampling from distribution mechanisms are suitable for non-continuous attributes *Gender*.

In step 2.2 adaptive sensitivity, we calculate adaptive sensitivity to add attribute-wise adaptive noise according to feature importance, such that $\Delta AS_{j_{i}}$ = $\Delta f_{j}$ − Δi. In this example, $\Delta AS_{j_{i}}$ for the most relevant attribute *Height* equals to 0.29 (=1 − 0.71). Furthermore, $\Delta AS_{j_{i}}$ for the most relevant attribute *Height* equals to 0.29 (=1 − 0.71), $\Delta AS_{j_{i}}$ for attribute *Gender* equals to 0.80 (=1 − 0.20), and $\Delta AS_{j_{i}}$ for the least relevant attribute *Age* equals to 0.91 (= 1 − 0.09).

In step 2.3 Synthesis, let us assume that we choose the Laplace mechanism as the output perturbation mechanism type and the Exponential mechanism as a sample from the distribution mechanism type. For both continuous attributes *Height* and *Age*, we add additive random noise drawn from Laplace distribution and scaled with corresponding $\Delta AS_{j_{i}}$ and ϵ values. In this example, privacy parameter ϵ is set to 1. Thus, random noise is represented as $Lap(\frac{0.29}{1})$ for *Height* attribute and $Lap(\frac{0.91}{1})$ for *Age* attribute. Furthermore, the amount of random noise generated for relevant attribute *Height* is smaller than the amount of random noise generated for the least relevant attribute *Age*.

For the categorical attribute *Gender*, we sample an element from a family-dependant distribution with random probability scaled by $\Delta AS_{j_{i}}$ equal to 0.80 and ϵ equal to 1. The probability of sampling a wrong element is smaller than the probability that it was scaled with record sensitivity. As a result, we obtain differentially private data $\hat{X}$ that guarantees privacy.

## 5. Experiments

To show the effectiveness of the proposed task-specific adaptive DP method, we do several experiments with various datasets, tasks of ML models, different differentially private mechanisms, and so on. Specifically, we evaluated the proposed DP method to answer the following questions:

- How does the performance of the proposed DP method change under different values of privacy parameter ϵ?
- How does the proposed DP method influence the utility of original data?
- Does the proposed DP method mitigate the modified AIA?
- Does the proposed DP method resolve the privacy–utility trade-off problem better than well-known differentially private data publishing methods?

To measure the performance of the proposed DP method, we run experiments on Windows 10, AMD Ryzen 5 3600 6-Core Processor, 16 Gb RAM, Python-3.8, and Jupyter Notebook.

To show the effectiveness of the proposed DP method under various datasets, we measured the performance of the proposed DP method and the existing methods under 9 real-life structured datasets, each of which consists of different types of data. Specifically, three datasets, i.e., Loan prediction [24], Car Insurance prediction [25], and Customer Segmentation [26] datasets, mainly consist of categorical attributes; other three datasets, i.e., Red Wine Quality [27], Boston Housing [28], and Breast Cancer diagnostic [29], mainly consist of continuous attributes; and the other three datasets, i.e., Adult Income [30], Bank Churn Modeling [31], and COVID-19 KCDC Patients [32], consist of a mixture of categorical and continuous attributes.

### 5.1. How Does the Performance of the Proposed DP Method Change under Different Values of Privacy Parameter ϵ?

To show the influence of privacy parameter ϵ on the performance of the proposed DP method, we generate differentially private data while varying values of privacy parameter ϵ from 0.01 to 10 in increments of 10. Furthermore, to show that the proposed method is data and model-agnostic, we trained 9 different ML models on 9 different datasets, respectively. Pair of ML models and the dataset are given according to each reference [24,25,26,27,28,29,30,31,32]. Specifically, we trained the Random Forest model with 50 estimators and a maximum depth of 5 for the Loan prediction dataset [24], AdaBoost Classifier with 50 estimators for Car Insurance prediction dataset [25], Gradient Boosting model with 60 estimators for Customer Segmentation dataset [26], Support Vector Machine with regularization parameter 1 for Red Wine Quality dataset [27], Linear Regression model for Boston Housing dataset [28], Naive Bayes Classifier for Breast Cancer diagnostic dataset [29], Logistic Regression model for Adult Income dataset [30], Artificial Neural Network that is a sequential model with 6 layers for Bank Churn modeling dataset [31], and Decision Tree Classifier model with a maximum depth of 10 and random state 10 for COVID-19 KCDC Patients dataset [32]. Note that, all models except Artificial Neural Network were implemented with Scikit-learn tools for Python [33]. We compare the performance of the given trained ML models under differentially private data from the proposed DP method and original data. Specifically, we train and fit the ML models mentioned above with the original data and denote their performance as baseline performance. Next, we train and fit another ML model with the same configurations but with the differentially private data generated from the proposed DP methods and compare the performance with the baseline performance.

Table 2 shows the accuracy of the proposed DP method for 9 ML models under different ϵ values. Here, differentially private test data are denoted as private data, and original test data are denoted as non-private data.

We observe that the accuracy of the proposed DP method is significantly lower under small ϵ values, while the accuracy of the proposed DP method is similar to baseline accuracy under large ϵ values for both private and non-private test data. These observations imply that smaller privacy parameter ϵ guarantees a strong guarantee of privacy and vice versa. For example, a large ϵ value equal to 10 is considered an impractical value due to a weak guarantee of privacy. For example, the proposed DP method for the private Loan dataset shows classification accuracy by as much as 71.0%, 85.1%, 89.0%, and 93.0% under ϵ values equal to 0.01, 0.1, 1 and 10, respectively, while the proposed DP method for the non-private Loan dataset shows classification accuracy by as much as 77.5%, 86.3%, 89.0%, and 93.7% under ϵ values equal to 0.01, 0.1, 1 and 10, respectively. We observe that the proposed DP method shows similar classification accuracy for private and non-private data under the corresponding ϵ value. We also observe that the proposed DP method shows high classification accuracy that is close to baseline accuracy under a small ϵ value equal to 1.

### 5.2. How Does the Proposed DP Method Influence the Utility of Original Data?

To evaluate the quality of differentially private data generated from the proposed DP method, we quantify the uncertainty of data using Shannon entropy. By measuring how much important information is lost from each attribute using Shannon entropy, we evaluate the influence of the proposed DP method on the utility of original data. We calculate the entropy of each feature in the dataset using the following as H=−sum(pk∗log(pk)), where pk is the probability of each value occurrence. Specifically, we compare the entropy of the original feature, the entropy of that feature after the proposed DP method, and the entropy of that feature after the naive DP method. Here, the term naive represents a single Laplace mechanism without consideration of feature importance or data type. To consider the influence of data types on the quality of differentially private data, we choose one dataset from each category of data type. That is, the loan prediction dataset for categorical data, the adult dataset for mixed data, and the red wine quality for numerical data are chosen.

**Figure 4:**
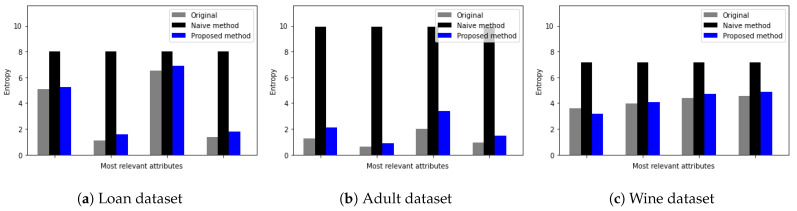
Entropy measure of most relevant attributes (**a**) Loan dataset [24], (**b**) Adult dataset [30], and (**c**) Red Wine quality dataset [27].

Figure 4 shows the Shannon entropy of the four most relevant features of three datasets. Here, the *x*-axis represents an attribute and the *y*-axis represents the Shannon entropy value. Furthermore, the grey color represents the entropy of the original attribute, black represents the entropy of the attribute with random noise from the naive DP method, and blue represents the entropy of the attribute with random noise from the proposed DP method. We observe that while the proposed DP method preserves the entropy of relevant features on the same level as the original data entropy, the naive DP method adds large uncertainty to the attribute. This is because the proposed DP method adds less noise to relevant features than the native DP mechanism.

For example, as shown in Figure 4a for the loan dataset, original entropy values for relevant attributes varied from 0.5 up to 6, while the entropy of relevant attributes with random noise added by the naive DP method increased up to 8 regardless of entropy values of original attributes. Furthermore, from Figure 4a–c, we observe that entropy values of relevant attributes with random noise added by the proposed DP method are similar to the original entropy values.

**Figure 5:**
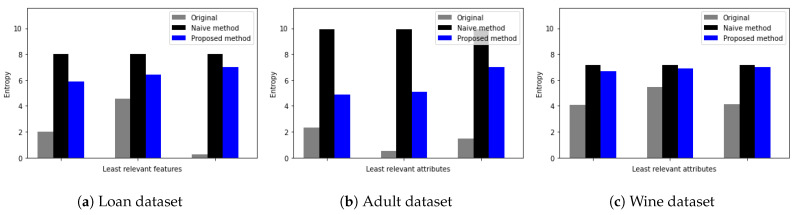
Entropy measure for least relevant attributes (**a**) Loan dataset [24], (**b**) Adult dataset [30], and (**c**) Red Wine quality dataset [27].

Figure 5 shows Shannon entropy for the three least relevant features in three datasets, respectively. Since the proposed DP method adds more noise to irrelevant features, we observe that the proposed DP method adds large uncertainty to attributes and thus, preserves the privacy of data. For example, as shown in Figure 5a, original entropy values for least relevant attributes vary from 0.05 up to 4.5, while the entropy of least relevant attributes with random noise added by the naive DP method increases up to 8 regardless of original attributes entropy value.

Furthermore, from Figure 5a–c, we observe that entropy values of least relevant attributes with random noise added by the proposed DP method are similar to entropy values with random noise added by the naive DP method. These observations imply that for the least relevant attributes, original entropy values are different from the entropy values of the proposed DP method and the naive DP method.

### 5.3. Does the Proposed DP Method Mitigate the Modified AIA?

**Figure 6:**
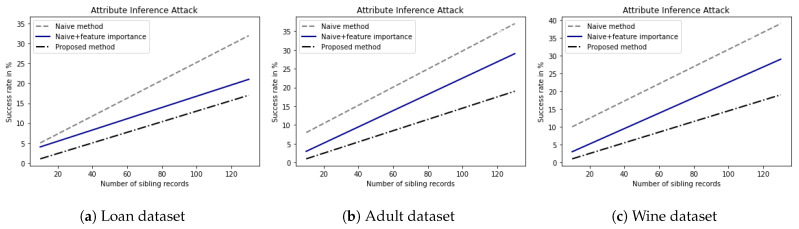
AIA success rate for (**a**) Loan dataset [24], (**b**) Adult dataset [30], and (**c**) Red Wine quality dataset [27].

To show the robustness of the proposed DP method under a privacy attack, we measured the performance of the proposed DP method under the modified AIA. We evaluated the success rates of the modified AIA for datasets with random perturbations generated by the naive DP method and the proposed DP method. Figure 6 shows the success rates of the modified AIA against the naive DP method and the proposed DP method for three representative datasets according to a number of sibling records. As shown in Figure 6a for the loan dataset, while the AIA success rate for the naive DP method increases from 5% up to 30% and AIA success rate for the naive DP method considering feature importance increases from 4% up to 17%, AIA success rate for the proposed DP method increases from 3% up to 14% with the increment of sibling records. As shown in Figure 6b for the adult dataset, while the AIA success rate for the naive DP method increases from 9% up to 35% and the AIA success rate for the naive DP method considering feature importance increased from 4% up to 25%, AIA success rate for the proposed DP method increases from 4% up 13% with the increment of sibling records.

These observations indicate that while the attack success rate against the naive DP method without considering the feature importance of the attribute is high, the naive DP method considering the feature importance of the attribute shows a low attack success rate. Furthermore, the attack success rate against the proposed DP method is the lowest. This is because the adaptive addition of random noise considering feature importance and type of data lowers the probability that the adversary generates high-quality sibling records and thus, lowers the success rate of the modified AIA.

### 5.4. Does the Proposed DP Method Resolve the Privacy–Utility Trade-Off Problem Better Than Well-Known Differentially Private Data Publishing Methods?

We compared the proposed DP method with well-known differentially private data publishing methods, such as PrivBayes [10], which is a representative MD-based method, and DPGAN [14], which is a representative GAN-based method. To measure privacy–utility trade-off curves under the influence of the modified AIA, we set the value of the number of sibling records to 100, which is one of the highest numbers to show the difference in AIA success rates.

As shown in Table 3, we compare the accuracy of the proposed DP method with well-known differentially private data publishing methods under two privacy parameter ϵ values, i.e., ϵ = 0.1 and ϵ = 1. For example, MD-based and GAN-based methods for loan dataset shows classification accuracy for private data by as much as 75.1% and 69.1%, respectively, while the proposed DP method shows higher classification accuracy by as much as 85.1% under ϵ equal to 0.1. Furthermore, MD-based and GAN-based methods for loan dataset shows classification accuracy for private data by as much as 84.7% and 67.3%, respectively, while the proposed DP method shows higher classification accuracy by as much as 89.0% under ϵ equal to 1.

From Table 3, we observe that the proposed DP method outperforms PrivBayes by as much as 4∼10% and DPGAN by as much as 10∼15% on average when privacy parameter ϵ equal 0.1. Furthermore, we observe that the proposed DP method outperforms PrivBayes by as much as 2∼6% and DPGAN by as much as 8∼12% on average when privacy parameter ϵ equal 1.

**Figure 7:**
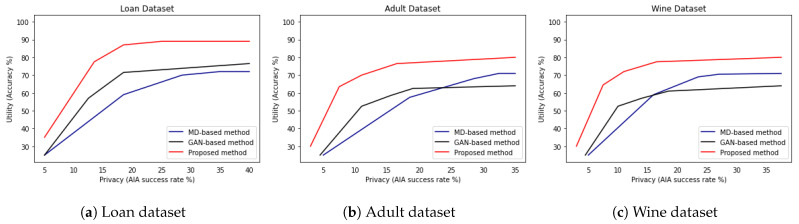
Privacy–utility trade-off for (**a**) Loan dataset [24], (**b**) Adult dataset [30], and (**c**) Red Wine quality dataset [27].

In Figure 7, we show privacy–utility trade-off curves of the proposed DP method and two well-known differentially private data publishing methods [10,14]. Let us note that we are interested in maximizing accuracy while minimizing the success rate of the modified AIA and thus, providing a high utility and privacy guarantee. In Figure 7a for the loan dataset, we observe that MD-based method [10] depicted by blue color shows the highest privacy–utility trade-off and GAN-based method [14] depicted by black color shows slightly lower privacy–utility trade-off, while the proposed DP method depicted by red color shows lowest privacy–utility trade-off. Furthermore, in Figure 7b for the adult dataset and Figure 7c for the red wine quality dataset, we observe that compared to both MD-based and GAN-based methods, the proposed DP method shows the lowest privacy–utility trade-off. These observations imply that the proposed DP method maximizes accuracy while minimizing the success rate of the modified AIA and thus, provides a high utility and privacy guarantee.

## 6. Conclusions

As a PPML technique that protects private data with sensitive information in ML, we proposed a new task-specific adaptive DP technique for structured data, while adaptively calibrating the amount and distribution of random noise applied to each attribute according to the feature importance for the specific tasks of ML models and different types of data, the proposed task-specific adaptive DP technique satisfies the model-agnostic property to be applied to a wide range of ML tasks and various types of data while resolving the privacy–utility trade-off problem. From experimental results under various datasets, tasks of ML models, different differentially private mechanisms, and so on, we evaluated the proposed DP method. Specifically, we answered how the performance of the proposed DP method varies under privacy parameter ϵ, how the utility of original data changes under the influence of the proposed DP method, how effective the proposed DP method is against the modified AIA and in resolving privacy–utility trade-off problem. For example, we showed that the data utility of the proposed DP method is higher than well-known differentially private data publishing methods while offering higher privacy guarantees against AIA.

## Figures and Tables

**Table 1:** Symbolic Notations.

Symbol	Description	Symbol	Description
*X*	Input data	ΔAS	Adaptive sensitivity
$X_{N}$	Neighbor dataset	*m*	Mechanism
*i*	Attribute	OP	Output perturbation
*j*	Record	SD	Sample from distribution
Δf	Sensitivity	ϵ	Privacy parameter
Δi	Feature importance	$\hat{X}$	Noisy data

**Table 2:** Accuracy of the proposed DP method for ML models under different ϵ values.

Data	Type	Dataset	Test Accuracy				
			ϵ = 0.01	ϵ = 0.1	ϵ = 1	ϵ = 10	Baseline
Private	Categorical	Loan [24]	71.0%	85.1%	89.0%	93.0%	94.5%
		Insurance [25]	58.1%	66.2%	76.0%	80.7%	83.2%
		Customer [26]	33.7%	41.0%	48.4%	50.6%	53.5%
	Continuous	Wine [27]	53.2%	68.9%	74.1%	76.7%	78.7%
		Housing [28]	49.2%	60.0%	72.3%	75.7%	78.0%
		Cancer [29]	62.8%	79.5%	90.1%	93.2%	96.4%
	Mixed	Adult [30]	50.2%	67.0%	72.6%	74.7%	77.0%
		Churn [31]	59.6%	75.3%	80.8%	84.1%	85.9%
		COVID-19 [32]	63.9%	74.0%	82.6%	85.8%	87.4%
Non-private	Categorical	Loan [24]	77.5%	86.3%	89.0%	93.7%	94.5%
		Insurance [25]	55.7%	67.1%	79.4%	82.4%	83.2%
		Customer [26]	30.4%	39.5%	49.0%	52.8%	53.5%
	Continuous	Wine [27]	51.3%	68.5%	75.8%	77.2%	78.7%
		Housing [28]	51.3%	63.7%	73.5%	77.8%	78.0%
		Cancer [29]	60.3%	77.8%	89.4%	97.0%	96.4%
	Mixed	Adult [30]	49.0%	67.1%	72.8%	73.7%	77.0%
		Churn [31]	64.0%	76.0%	82.1%	85.0%	85.9%
		COVID-19 [32]	59.2%	77.3%	83.4%	86.9%	87.4%

**Table 3:** Comparison of performance with conventional methods.

Dataset	Method	Test Accuracy			
		ϵ = 0.1		ϵ = 1	
		Private	Non-Private	Private	Non-Private
Loan [24]	MD-based [10]	75.1%	76.3%	84.7%	83.1%
	GAN-based [14]	69.1%	70.6%	67.3%	64.9%
	Proposed	85.1%	86.3%	89.0%	90.4%
Insurance [25]	MD-based [10]	62.4%	65.2%	70.3%	77.4%
	GAN-based [14]	52.7%	50.2%	58.0%	54.2%
	Proposed	66.2%	67.1%	76.0%	79.4%
Customer [26]	MD-based [10]	37.8%	35.0%	48.3%	46.3%
	GAN-based [14]	34.0%	35.1%	38.1%	37.6%
	Proposed	41.0%	39.5%	52.4%	49.0%
Wine [27]	MD-based [10]	63.8%	63.7%	69.9%	73.0%
	GAN-based [14]	55.0%	74.3%	59.1%	67.1%
	Proposed	68.9%	68.5%	74.1%	75.8%
Housing [28]	MD-based [10]	55.3%	48.9%	67.9%	69.4%
	GAN-based [14]	45.5%	44.9%	45.9%	57.1%
	Proposed	60.0%	63.7%	74.1%	75.8%
Cancer [29]	MD-based [10]	74.2%	70.4%	84.7%	77.5%
	GAN-based [14]	65.3%	60.8%	54.9%	64.1%
	Proposed	79.5%	77.8%	90.1%	89.4%
Adult [30]	MD-based [10]	64.8%	59.0%	70.2%	73.0%
	GAN-based [14]	55.3%	62.6%	62.3%	68.1%
	Proposed	67.0%	67.1%	72.6%	72.8%
Churn [31]	MD-based [10]	70.5%	69.3%	78.0%	81.3%
	GAN-based [14]	60.7%	62.3%	71.6%	74.9%
	Proposed	75.3%	76.0%	80.8%	82.1%
COVID-19 [32]	MD-based [10]	67.1%	67.8%	75.6%	79.7%
	GAN-based [14]	59.6%	64.2%	67.2%	70.9%
	Proposed	74.0%	77.3%	82.6%	83.4%

## Data Availability

The data presented in this study are openly available, and the reference numbers are [24,25,26,27,28,29,30,31,32].
